# Prioritizing persons deprived of liberty in global guidelines for tuberculosis preventive treatment

**DOI:** 10.1371/journal.pmed.1004288

**Published:** 2023-10-03

**Authors:** Aditya Narayan, Argita D. Salindri, Salmaan Keshavjee, Monde Muyoyeta, Kavindhran Velen, Zulma V. Rueda, Julio Croda, Salome Charalambous, Alberto L. García-Basteiro, Sheela V. Shenoi, Crhistinne C. M. Gonçalves, Liliane Ferreira da Silva, Lia G. Possuelo, Sarita Aguirre, Gladys Estigarribia, Guillermo Sequera, Louis Grandjean, Lily Telisinghe, Michael E. Herce, Fernanda Dockhorn, Frederick L. Altice, Jason R. Andrews

**Affiliations:** 1 Division of Infectious Diseases and Geographic Medicine, Stanford University School of Medicine, Stanford, California, United States of America; 2 Department of Global Health and Social Medicine, Harvard Medical School, Boston, Massachusetts, United States of America; 3 Division of Global Health Equity, Department of Medicine, Brigham and Women’s Hospital, Boston, Massachusetts, United States of America; 4 Centre for Infectious Disease Research in Zambia (CIDRZ), Lusaka, Zambia; 5 Implementation Division, The Aurum Institute, Johannesburg, South Africa; 6 Department of Medical Microbiology and Infectious Diseases, University of Manitoba, Winnipeg, Canada; 7 Research Department, School of Medicine, Universidad Pontificia Bolivariana, Medellin, Colombia; 8 School of Medicine, Federal University of Mato Grosso do Sul, Campo Grande, Brazil; 9 Department of Medicine, Section of Infectious Diseases, Yale School of Medicine, New Haven, Connecticut, United States of America; 10 Oswaldo Cruz Foundation, Campo Grande, Brazil; 11 Wits School of Public Health, Johannesburg, South Africa; 12 ISGlobal, Hospital Clínic, Universitat de Barcelona, Barcelona, Spain; 13 Manhiça Health Research Center, Maputo, Mozambique; 14 Centro de Investigación Biomédica en Red de Enfermedades Infecciosas (CIBERINFEC), Barcelona, Spain; 15 Department of Life Sciences, Santa Cruz do Sul University, Santa Cruz do Sul, Brazil; 16 National Tuberculosis Control Program, Ministry of Public Health and Social Welfare (MSPyBS), Asunción, Paraguay; 17 Instituto Regional de Investigación en Salud, Caaguazú, Paraguay; 18 Department of Public Health, Facultad de Ciencias Médicas, Universidad Nacional de Asunción, Asunción, Paraguay; 19 Department of Infection, Immunity and Inflammation, Institute of Child Health, University College London, London, United Kingdom; 20 London School of Hygiene and Tropical Medicine, London, United Kingdom; 21 Institute for Global Health and Infectious Diseases, University of North Carolina at Chapel Hill, Chapel Hill, North Carolina, United States of America; 22 Ministry of Health, Health and Environmental Surveillance Secretariat, General Coordination for Tuberculosis, Endemic Mycoses and Non-Tuberculous Mycobacteria Surveillance, Brasília, (DF) Brazil

## Abstract

In this Policy Forum piece, Aditya Narayan and colleagues discuss the challenges and opportunities for tuberculosis preventive treatment in carceral settings.

**Summary points**

- Persons deprived of liberty (PDLs) are disproportionately impacted by tuberculosis, with high incidence rates and often limited access to diagnostics, treatment, and preventive measures.
- The World Health Organization (WHO) expanded its recommendations for tuberculosis preventive treatment (TPT) to many high-risk populations, but their guidance does not include PDL, and most low- and middle-income countries do not routinely provide TPT in prisons.
- Recent studies demonstrate high acceptability and completion rates of short-course TPT regimens in jails and prisons; costs of these regimens have been markedly reduced through international agreements, making this an opportune for further expanding their use.
- We argue that PDL should be a priority group for TPT in national guidelines and discuss implementation considerations and resource needs for TPT programs in carceral facilities.
- Scaling access to TPT for PDL is important for reducing disease and transmission in this population; it is also critical to advancing an equitable response to tuberculosis.

Tuberculosis continues to be a leading cause of illness and death globally, with greater than 10 million people becoming ill with tuberculosis and 1.6 million deaths estimated to occur each year [1]. The disease disproportionately affects impoverished and socially marginalized populations, with persons deprived of liberty (PDLs) in closed carceral settings having among the highest incidence [2]. Globally, over 10.7 million individuals are held in penal institutions, and several times as many people pass into and out of carceral facilities each year [3]. A recent meta-analysis reported that tuberculosis incidence among PDL was over 10 times higher than that of their surrounding communities [2]. Carceral settings have long been recognized as high-risk settings for tuberculosis disease and transmission. Densely populated by individuals with multiple social and biological risk factors, prison environments are ideal for tuberculosis transmission. Additionally, prisons are severely under resourced, with limited healthcare services, frequently facing shortages of medical staff, and insufficient diagnostic capacity for tuberculosis and related medical comorbidities. Uncontrolled tuberculosis transmission in prisons may amplify epidemics in their surrounding communities, undermining national and regional targets for tuberculosis control [4–6].

In response to the growing evidence reporting the disproportionate burden of tuberculosis faced by PDLs, the World Health Organization (WHO) updated its guidance in 2021 to strongly recommend systematic screening for tuberculosis disease in prisons [7]. Such guidance is critical to ensuring policymakers invest adequately in tuberculosis diagnosis and treatment in carceral facilities [8]. WHO guidelines for tuberculosis preventive treatment (TPT) recently expanded the scope of priority groups for this intervention, however, do not clearly recommend its use in carceral settings [9]. The guidelines only briefly mention prisoners among other risk groups in whom tuberculosis infection testing and treatment “may be considered,” which is a “conditional recommendation” and insufficient to promote policy change. Specific guidance addressing the unique considerations around testing and treatment in carceral settings remain unavailable. Consequently, very few countries with high tuberculosis burden routinely provide tuberculosis infection testing and treatment in prisons. Yet, the case for expanding TPT to PDL has never been stronger. Global estimates for the incidence of tuberculosis in prisons are available for the first time in 2023, documenting a large and growing burden in many regions [10]. Recent studies demonstrate that short-course TPT regimens are associated with high acceptance and completion rates in prisons. A 2022 negotiated agreement markedly reduced the cost of short-course regimens in low- and middle-income countries (LMICs), reducing a key financial barrier [11]. Here, we review and contextualize these developments, discussing their implications along with policy and implementation considerations to inform the design of TPT programs in prisons.

## Screening for tuberculosis infection and disease in prisons

Recently published global estimates, leveraging data from more 150 countries, estimated that 125,000 people develop tuberculosis disease in prisons each year, while only 53% are detected and notified [10]. Estimated disease incidence was extremely high in all WHO regions, ranging from 793 cases per 100,000 in the Eastern Mediterranean region to 2,242 cases per 100,000 in the Africa region. In the Americas, the estimated number of tuberculosis cases in prisons has tripled since 2000, amid rising incarceration rates. While fewer data are available concerning the incidence of tuberculosis infections, a recent meta-analysis documented a very high pooled incidence of 15 people acquiring infection per 100 person-years [2]. These high rates of infection point towards an opportunity prevent tuberculosis disease and subsequent transmission using TPT.

A key question for programs providing testing and treatment of tuberculosis infection is the timing of screening. In high tuberculosis–burden communities, and among previously incarcerated individuals, the prevalence of tuberculosis infection is often high, supporting an approach of screening at entry or reentry. By contrast, among individuals incarcerated for the first time in communities with low or moderate tuberculosis incidence, prevalence may be low at entry. For example, a study in Brazil found that <10% of individuals had positive tuberculin skin tests at the time of first incarceration but then incurred a >20% annual risk of infection during incarceration [12]. Taken together, these data suggest that screening for tuberculosis infection should be performed at prison entry and periodically to assess for incident infections. In settings with high transmission rates, twice annual screening for incident infections may be needed, with the latter serving to identify recently infected individuals in time to initiate TPT and prevent disease.

Testing for tuberculosis infection may be performed by tuberculin skin tests (TSTs) or interferon gamma release assays (IGRAs); the latter are typically more expensive and require phlebotomy and laboratory equipment. Both approaches, however, confer different advantages with respect to specificity and sensitivity, as well as the need for follow-up. In some high tuberculosis–burden settings, including prisons, programs have elected to forego testing for tuberculosis infection and directly offer preventive treatment to all high-risk individuals [13]. The rationale for this may be multifold: (1) in high transmission settings, the majority of individuals will harbor tuberculosis infection, and the risk of a false negative IGRA/TST is greater, particularly among those with HIV in whom TST has reduced sensitivity; (2) TPT may prevent infections while it is taken, as some clinical trial evidence suggests [14,15]; (3) historical studies in high transmission settings suggested that individuals with negative TSTs were at highest risk of tuberculosis, presumably due to greater susceptibility to initial infection [16]; and (4) the costs of TPT are lower than IGRA testing. Although there have been shortages in tuberculin for TST, new tuberculosis antigen-based skin tests may soon mitigate this problem. The innovation of providing “community-wide” preventive treatment in settings with very high transmission rates is not a new one; some of the earliest isoniazid trials found that community-wide use of preventive treatment was efficacious for individuals with and without infection [17]. Such strategies need to be balanced against risks of adverse events, particularly in populations with medical comorbidities that elevate risk of drug toxicities. Testing for tuberculosis infection must be combined with screening for tuberculosis disease, ideally using both radiography and rapid molecular diagnostics, to identify individuals who require treatment. Many prisons, particularly in LMICs, lack functioning equipment and personnel for performing chest radiography. New, portable chest radiography systems, paired with automated interpretation software, hold promise for making radiography-based screening for disease more accessible in prisons [18,19].

## Tuberculosis preventive treatment regimens in carceral settings

With respect to TPT regimens, 6 to 9 months of daily isoniazid (INH) was historically the most commonly used regimen in carceral settings. The effectiveness of this strategy in these settings has been limited, however, due to low treatment acceptance and completion rates. A systematic review of INH-based regimens delivered in carceral settings found a median completion rate of just 44% due to patient and system-level factors [20]. Equally efficacious short-course regimens, including a 3-month (12 weekly dose) regimen of high-dose isoniazid and rifapentine (3HP), have led to markedly improved completion rates in carceral settings compared with 6 or 9 months of isoniazid [21,22]. Two recent studies illustrate successful models for short-course–based TPT in high-burden LMIC prisons. In Malawi and Pakistan, 3HP acceptance in prisons exceeded 95% and completion rates were over 85%, with low rates of adverse events [13,23].

The cost of rifapentine remains one of the major obstacles to adoption and scale-up of short-course TPT regimens in LMICs. UNITAID and its partners brokered a 70% price reduction in 3HP in 2022, with the entire course now available for $14.25 [11]. Such innovations should accelerate 3HP scale-up, yet the manufacturer of this off-patent medication has not made rifapentine routinely available to many countries [24]. Other short-course regimens are also recommended and associated with improved completion rates compared with 6 or 9 months of INH, but, to our knowledge, there are minimal data concerning their use in prisons and jails. These include a 3-month daily regimen of isoniazid and rifampicin and a 4-month daily regimen of rifampicin. Studies are needed to compare adherence, completion, and tolerability, as well as implementation feasibility in prisons, for these daily versus weekly short-course regimens. “Ultra-short-course” regimens, such as the 1-month regimen of daily INH and rifapentine (1HP), appeared noninferior to INH-based regimens among people living with HIV, which has led to a WHO recommendation for their use in this population [25]. Trials assessing 1HP’s effectiveness in individuals without HIV are ongoing. These shorter regimens may increase the likelihood that TPT is completed prior to leaving prisons, which has been a problem with longer regimens.

## Challenges and opportunities for tuberculosis preventive treatment in carceral settings

One of the principal challenges to effective delivery of TPT in carceral settings is treatment continuity among individuals leaving the setting, due to interinstitutional transfer or release to the community, which historically resulted in low linkage to care and completion rates [26]. Short-course regimens increase the chance that treatment can be completed before release but have not completely mitigated this problem. Strategies to improve preventive treatment completion have included education, transitional care clinics, and small financial incentives, which have had varying success [27].

In most countries, PDL have higher rates of psychiatric and substance use disorders and related comorbidities, including HIV and chronic hepatitis B virus (HBV) and hepatitis C virus (HCV) infections [28]. These comorbidities are risk factors for adverse events, particularly drug-induced hepatitis from longer regimens, and treatment noncompletion. Testing and treatment for HIV, HBV, and HCV should be integrated with TPT programs, and those with HIV and/or viral hepatitis coinfection may require selection of regimens with lowest risk of liver injury and drug–drug interactions, along with closer monitoring for hepatoxicity. In patients with comorbidities placing them at risk of adverse events from TPT, counseling concerning risks and benefits should be undertaken as part of shared decision-making about whether to initiate treatment. Additionally, substance use and psychiatric disorders should be treated concomitantly; this may include medications for opioid use disorder (MOUDs) for individuals with opioid use disorders. Studies suggest that MOUD substantially improves each step of both the HIV and tuberculosis treatment cascades and have the potential to serve as a bridge to treatment completion after release [29,30]. Because rifampin and rifapentine are potent inducers of cytochrome P450 enzymes and can affect metabolism of opioids and opioid agonists, clinicians should monitor closely during therapy to assess the need for medication adjustments.

Although treatment of tuberculosis infection has been standard of care in the United States since the early 1960s and the scope of its recommended use was recently broadened by WHO, there continues to be concern about implementing TPT programs among PDL (Table 1). One serious concern is that reinfection following treatment will compromise the effectiveness of the intervention in high-transmission settings, as no tests exist to measure reinfection. Studies among South African gold miners revealed a rapid rebound in tuberculosis incidence within 6 months of completion of TPT [31], and similar patterns could be seen among people who remain incarcerated and exposed to a high force of infection. We note that many individuals in high tuberculosis–burden countries are incarcerated for short sentences, often less than 2 years, such that the exposure to reinfection may be lower. For those who remain incarcerated, prolonged or pulsed preventive treatment may be indicated, though data are mixed. A randomized trial of prolonged (36-month) isoniazid TPT was more effective than 6 months of isoniazid among adults living with HIV in Botswana [15]. Other trials in persons living with HIV found durable effectiveness of 6 months of isoniazid and no superiority of longer durations [32,33]. In a trial conducted in communities in South Africa, Ethiopia, and Mozambique, a repeat round of TPT at 12 months did not confer additional benefits for individuals receiving antiretroviral therapy though the incidence and force of infection in those communities are lower than that of many prisons [34]. Further studies should investigate the potential for continuous or pulsed preventive treatment in high tuberculosis–burden prisons. Finally, TPT programs should be implemented alongside intensive active case finding programs, to reduce transmission and risk of reinfection.

**Table 1:** Challenges, needs, and opportunities for implementing tuberculosis preventive treatment program in carceral settings.

Challenges	Proposed recommendations	Potential obstacles	Research needs	Expected impact
Identifying priority groups for TPT	Screen for tuberculosis disease and infection at entry and at least annually	Inadequate resources and health personnel in carceral settings in many high TB–burden countries	Identification of efficient screening algorithms for TB infection and disease	Evidence to guide the mobilization of public health resources for prison-based TB preventive efforts Identification of screening algorithms that are effective, low cost, high yield, and scalable Identification of subgroups that are at high risk to develop TB disease during or following incarceration to prioritize in TPT programs
	Guidance for those previously treated with TB/TPT regimens	High rates of reexposure to TB and lack of biomarkers to identify reinfection	Trials of periodic preventive treatment for individuals with continued TB exposure Biomarkers to identify individuals at risk of TB progression	
	Identification of TB risk factors in the context of carceral settings (i.e., illicit drug use, smoking, HIV, hepatitis B and C)	Lack of integrated health services for common TB-related coinfections and comorbidities Carceral health services lack resources for treatment of viral hepatitis and substance use disorders	Evaluation of integrated testing and treatment for TB infection and key comorbidities	
Determining an effective and contextually appropriate TPT delivery scheme	Implement the use of shorter TPT regimens (e.g., 3HP)	Comorbid liver disease (e.g., alcohol-related liver disease, viral hepatitis) and drug–drug interactions with opioid agonists	Comparative studies of short-course regimens, including in people with risk factors for hepatotoxicity	Identification of optimal TPT regimens according to incarceration duration and medical comorbidities Alternative of TPT implementation and delivery models aiming to result in highest completion rates within and beyond carceral settings
	Establish referral and linkage networks to ensure continuity for TPT and other medical care following return to community	Limited communication and medical record sharing between carceral and community health services Barriers to accessing to health services in community settings, including economic hardship and fear of stigma or reporting to police Lack of access to illicit drug use treatment in community settings	Implementation science studies to identify optimal strategies to ensure linkage to care and treatment completion for individuals leaving prisons during treatment for TB infection	

HIV, human immunodeficiency virus; TB, tuberculosis; TPT, tuberculosis preventive treatment; 3HP, 3 month regimen of weekly isoniazid and rifapentine.

In many settings, the burden of rifampicin-resistant (RR) or multidrug-resistant (MDR) tuberculosis is higher in prisons than in the surrounding communities, with several studies identifying prisons as important drivers of MDR/RR tuberculosis in the broader population [4,35]. While observational studies suggest that fluoroquinolones may be effective as preventive treatment for MDR tuberculosis [36,37], data from randomized trials are still lacking. Randomized trials are currently underway to test the effectiveness of TPT regimens among contacts of people with MDR tuberculosis. While awaiting these data, screening using rapid molecular diagnostics for rifampicin resistance should be performed for contacts of individuals with RR tuberculosis or in prisons with a high prevalence of RR among individuals with newly identified tuberculosis; preventive treatment using fluoroquinolone-based regimens may reduce risk of tuberculosis among these individuals [36].

## The path forward: Updating guidelines and national policies for tuberculosis preventive treatment

Preventive treatment remains a crucial but underutilized intervention for reducing the high tuberculosis incidence in carceral settings, particularly in LMICs where the greatest burden occurs. Further, given the dynamic nature of incarceration and high incidence of disease among individuals following release, prevention of tuberculosis in prisons may have outsized benefits for their surrounding communities. Data demonstrating high treatment completion rates for short-course regimens in these settings, together with reductions in the price of rifapentine, make this an opportune moment for expanding access to preventive treatment for PDL. In the absence of international guidance, most countries will not incorporate TPT for PDL into national guidelines and financially prioritize its implementation. Updating and upgrading recommendations for TPT use among PDL, including provision of specific guidance around approaches to testing for infection, screening for disease, and management of medical comorbidities, could provide the foundation for national tuberculosis programs to increase focus on and resources for this population. Given the extraordinarily high incidence of tuberculosis in prisons, such efforts are likely to reduce disparities and advance health equity.

## Abbreviations

HBV: hepatitis B virus
HCV: hepatitis C virus
IGRA: interferon gamma release assay
LMIC: low- and middle-income country
MDR: multidrug-resistant
MOUD: medication for opioid use disorder
PDL: person deprived of liberty
RR: rifampicin-resistant
TPT: tuberculosis preventive treatment
TST: tuberculin skin test
WHO: World Health Organization
